# Quantitative Structure–Toxicity Relationship in Bioactive Molecules from a Conceptual DFT Perspective

**DOI:** 10.3390/ph15111383

**Published:** 2022-11-10

**Authors:** Ranita Pal, Shanti Gopal Patra, Pratim Kumar Chattaraj

**Affiliations:** 1 Advanced Technology Development Centre, Indian Institute of Technology Kharagpur, Kharagpur 721302, India; 2 Department of Chemistry, Indian Institute of Technology Kharagpur, Kharagpur 721302, India

**Keywords:** conceptual density functional theory, electrophilicity index, hydrophobicity, QSAR, multiple linear regression

## Abstract

The preclinical drug discovery stage often requires a large amount of costly and time-consuming experiments using huge sets of chemical compounds. In the last few decades, this process has undergone significant improvements by the introduction of quantitative structure-activity relationship (QSAR) modelling that uses a certain percentage of experimental data to predict the biological activity/property of compounds with similar structural skeleton and/or containing a particular functional group(s). The use of machine learning tools along with it has made life even easier for pharmaceutical researchers. Here, we discuss the toxicity of certain sets of bioactive compounds towards *Pimephales promelas* and *Tetrahymena pyriformis* in terms of the global conceptual density functional theory (CDFT)-based descriptor, electrophilicity index (*ω*). We have compared the results with those obtained by using the commonly used hydrophobicity parameter, log*P* (where *P* is the *n*-octanol/water partition coefficient), considering the greater ease of computing the *ω* descriptor. The Human African trypanosomiasis (HAT) curing activity of 32 pyridyl benzamide derivatives is also studied against *Tryphanosoma brucei*. In this review article, we summarize these multiple linear regression (MLR)-based QSAR studies in terms of electrophilicity (*ω*, *ω*^2^) and hydrophobicity (log*P*, (log*P*)^2^) parameters.

## 1. Introduction

With the progress of modern science, especially biochemistry and synthetic organic chemistry, the field of drug discovery has witnessed huge advances in the use of statistical approaches. The process of clinical drug screening has always been an exhaustive and time-consuming process. Thus, the involvement of statistics and computational techniques in predicting the activity of potential drug molecules with structural similarities has greatly accelerated the process.

In this regard, quantitative structure-activity relationship (QSAR) modeling that uses a certain percentage of experimental data plays a crucial role to predict the biological activity/property of compounds with similar structural skeletons and/or containing a particular functional group(s) [1,2,3,4,5,6]. They are applicable in the fields of molecular modelling, drug discovery, eco-toxicology, antitumor treatment, etc. Hansch et al. [7] in 1962 first reported a QSAR-based study through the correlation between *n*-octanol/water partition coefficient and biological activity exhibited by those compounds. Many scientists have since followed in his footsteps and reported several such experimental and theoretical studies that have shaped the field of modern QSAR [8,9,10,11,12,13,14,15,16,17,18,19,20,21,22,23,24,25,26,27,28,29,30,31,32,33,34,35,36].

Toxicity of organic compounds is measured in terms of parameters like pLC_50_, pIC_50_, and pIGC_50_ which essentially measure the negative logarithm of the concentration of the toxin needed to kill half of the target population. These measures of toxicity have been extensively used in QSAR studies [37,38,39,40,41,42]. In toxicology studies, the aforementioned concentration depends on the toxicokinetic and toxicodynamic processes in a linear fashion. A library of descriptors, namely, quantum chemical and electronic energy descriptors, thermodynamic potentials, hydrophobicity, shape, topology, etc., have been used in QSAR-based studies [43]. Several global and local reactivity parameters are available within the purview of conceptual density functional theory (CDFT) that act as effective descriptors for predicting biological and toxicological activities [44,45,46,47,48,49]. Electrophilicity index (*ω*) is one such important descriptor that quantifies the electro-deficient nature of a molecule [50,51]. It has been previously utilized in predicting the biological activity (in terms of relative binding affinity values) of testosterone and estrogen derivatives [40], and the toxicity of benzidine [52] and polychlorinated biphenyls [38], to name a few. In recent times, machine learning is also becoming an integral part of drug design and QSAR studies in general [53,54,55,56,57].

This article provides a concise discussion on the toxicity of certain sets of bioactive compounds in terms of the global and local electrophilicity indices, and the simple yet effective methods utilized in developing the models.

## 2. Theoretical Background

According to Hansch [58,59], any correlation drawn between the biological activity of any system and its physicochemical properties ideally includes a steric part, a hydrophobic part, and an electronic part, to model a proper mathematical representation of the bioactivity. Now, the percentages of these three will vary depending on the mechanism, the receptor site, the mode of action, and several other factors. The hydrophobicity (or lipophilicity) parameter has gained a lot of importance in its usage as a descriptor owing to the fact that it can effectively describe the protoplasmic environment within a biosystem. However, using only the hydrophobic parameter is not enough in several types of mechanisms. Both receptor- and non-receptor-mediated toxicological reactions can occur either via covalent or noncovalent mechanisms. The latter form is especially important in aqueous toxicity, where the inclusion of the electronic parameter is required to produce a statistically relevant SAR model since hydrophobicity alone cannot describe the narcotic properties of polar chemicals. Thus, evaluating the electronic state of a compound is extremely useful in the prediction of its biological/toxicological properties, especially in reaction mechanisms driven by electrophile–nucleophile interactions. In this regard, we have resorted to CDFT-based chemical concepts.

For a system containing *N*-electrons, chemical reactivity parameters such as electronegativity (*χ*) [60,61] and chemical hardness (*η*) [62] are obtained within the domain of CDFT [63]. The former describes the ability of an atom in a molecule to attract bonded electrons towards itself and is perhaps the most fundamental descriptor required for the analysis of chemical activity. In DFT, this parameter is defined as the first derivative of the total energy (*E*) with respect to the total number of electrons (*N*) while the external potential ($v\left(\vec{r}\right)$) is kept constant. Parr [64] made a connection of this formulation with the negative of chemical potential (*µ*) which describes the escaping tendency of the electron cloud (see Equation (1)). Over the years many scales of electronegativity have been developed, such as those provided by Pauling [60,65], Mulliken [66], Allred-Rochow [67], etc. The second derivative of *E* with respect to *N* at constant $v\left(\vec{r}\right)$, on the other hand, describes the chemical hardness (*η*) of a system. This, by extension to Equation (1), becomes the first derivative of *µ* with respect to *N* (see Equation (2)). These definitions of electronegativity, chemical potential, and hardness lead up to the description of the electrophilicity index (*ω*) [64,68,69]. It first originated in the field of organic chemistry by Ingold’s [70,71] classification of organic chemical reactions in two groups: the electron-deficient species (electrophiles) characterized by their electrophilicity, and electron-rich species (nucleophiles) characterized by their nucleophilicity. These qualitative descriptions came long before any mathematical representation of electrophilicity was known. Finally, after Maynard’s [72] qualitative description of *ω* as the ratio between the square of electronegativity and hardness, Parr et al. [51] gave their quantitative description and defined it as the “electrophilic power” of a ligand (see Equation (3)), comparing it to the definition of power in classical electrostatics.

(1) $$\chi =-\mu ={\left(\frac{\partial E}{\partial N}\right)}_{v\left(\vec{r}\right)}$$

(2) $$\eta ={\left(\frac{\partial^{2}E}{\partial N^{2}}\right)}_{v\left(\vec{r}\right)\;}={\left(\frac{\partial \mu }{\partial N}\right)}_{v\left(\vec{r}\right)}$$

(3) $$\omega =\frac{\mu^{2}}{2\eta }=\frac{\chi^{2}}{2\eta }$$

During computation, we evoke the finite difference approximation [73] where the ∂N is approximated as the transfer of one electron to or from the neutral system and the ∂E becomes the corresponding energy change (*E_N_*_−1_ − *E_N_* or *E_N_* − *E_N_*_+1_). This way, the differential equation can be transformed into a set of algebraic equations. The self-consistent field (SCF) energy of the neutral system (with *N* electrons) and total single point energies of the cationic (*E_N_*_−1_) and anionic (*E_N_*_+1_) systems are calculated to obtain the ionization potential (I) and electron affinity (A) of the system as follows:

(4) $$I=E_{N-1}-E_{N}$$

(5) $$A=E_{N}-E_{N+1}$$

Thus, the hardness, chemical potential, and electronegativity are approximated as

(6) η ≈ I−A

(7) $$µ=-\chi \approx -\frac{1}{2}\left(I+A\right)$$

Alternatively, to avoid high computational cost, we sometimes employ Koopmans’ theorem [74] to calculate *I* and *A* as follows (*E*_HOMO_ and *E*_LUMO_ are the energies of the highest occupied and lowest unoccupied orbitals, respectively)

(8) $$I=-E_{HOMO}$$

(9) $$A=-E_{LUMO}$$

Equations (6) and (7) then transform to the following

(10) $$\eta \approx E_{LUMO}-E_{HOMO}$$

(11) $$\mu =-\chi \;\approx \frac{1}{2}\left(E_{HOMO}+E_{LUMO}\right)$$

The electrophilicity index is then evaluated using the values of *μ* and *η* following Equation (3). Although the global reactivity indices are fundamentally related to the energies of HOMO and LUMO energies, they help bring out different aspects of the electronic structure of the systems [75,76].

## 3. Methodology

**Figure 1:**
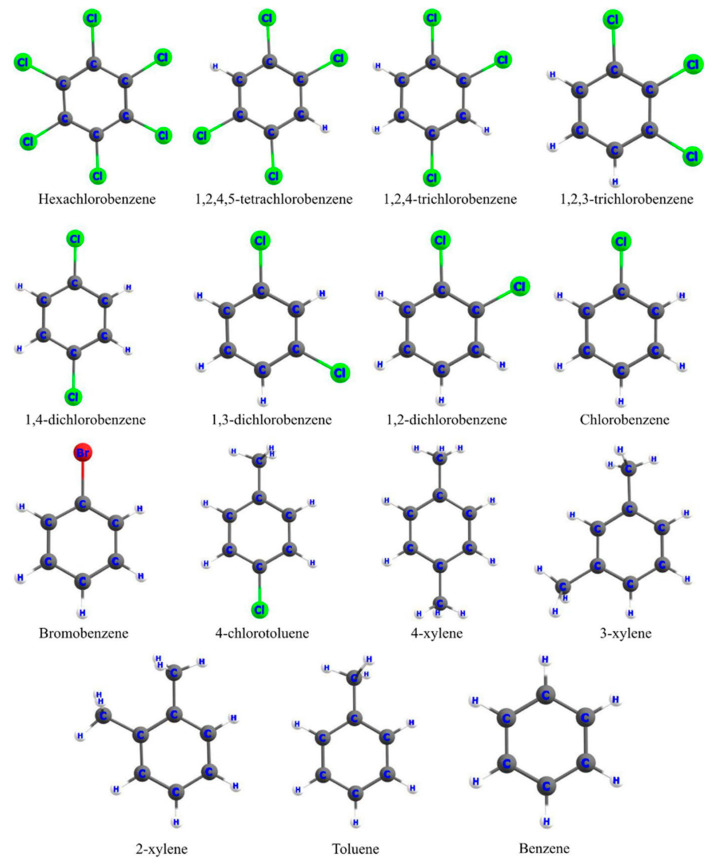
Geometries of the substituted benzene derivatives optimized at the B3LYP/6-31G(d) level of theory (reprinted from ref. [56] with permission from John Wiley & Sons. © 2018 John Wiley & Sons A/S).

First and foremost, energy minimization by way of geometry optimization is performed on all the compounds present in the dataset, viz., a set of 15 benzene derivatives for studying the toxicity against *Pimephales promelas* (Figure 1) [56], datasets of polychlorinated dibenzofurans (PCDFs) (Table S1) and polychlorinated biphenyls (PCBs) (Table S2) against radio-labeled tetrachlorodibenzo-p-dioxin (TCDD) [77], a set of 252 aliphatic compounds (comprising alcohols, esters, acids, aldehydes, ketones, and amines; Table S3) against *Tetrahymena pyriformis* [78], and a set of 32 pyridyl benzamides (Table S4) against *Trypanosoma brucei* [79]. The levels of theory used for computation of the above datasets are B3LYP/6-31G(*d*) (for the first two datasets), HF/6-311G**, and HF/6-31G(*d*) for the third and fourth datasets, respectively. The geometry optimization is followed by a frequency calculation on the compounds at the respective levels of theory to ensure the absence of any imaginary frequency. These computations are performed using the Gaussian 16 program package [80]. Relevant CDFT descriptors are then evaluated with the help of the equations provided in the “Theoretical background” section. We have then employed the multiple linear regression (MLR) and neural network (NN) methods to construct statistically relevant, robust QSAR models for predicting toxicological/biological activities of several datasets.

The general formula of any QSAR model is as follows:

Activity/Property/Toxicity = *f* (physicochemical properties)

= *a*_0_ + *a*_1_*x*_1_ + *a*_2_*x*_2_ + *a*_3_*x*_3_ + … (12)

where *x_n_* are the descriptors and *a_n_* are their corresponding coefficients. 

In our cases, the mathematical descriptors for toxicity are pLC_50_, pIC_50_, and pIGC_50_ acting as the dependent variables, whereas the global and local electrophilicity indices and hydrophobicity are the independent variables.

The efficacy of the constructed model is determined by some statistical parameters, viz., coefficient of determination (*R*^2^), adjusted *R*^2^ (*R*^2^*_adj_*), standard deviation (*SD*)

(13) $$R^{2}=1-\frac{\sum {\left(Y_{obs}-Y_{calc}\right)}^{2}}{\sum {\left(Y_{obs}-{\bar{Y}}_{obs}\right)}^{2}}$$

(14) $$R_{adj}^{2}=\frac{\left(N-1\right)*R^{2}-p}{N-1-p}$$

(15) $$SD=\sqrt{\frac{\sum {\left(Y_{obs}-Y_{calc}\right)}^{2}}{N-1-p}}$$

where *Y_obs_* and *Y_calc_* are the experimental and predicted dependent variables, respectively (in our case, toxicities), *N* is the total number of observables, and *p* denotes the number of descriptors used in the model.

### 3.1. Multiple Linear Regression (MLR)

The simple yet most widely accepted method, multiple linear regression (MLR) [81,82,83,84], uses regression coefficient or the coefficient of determination (*R*^2^) and standard deviation (SD) as its statistical metrics to judge the efficacy of the generated QSAR model. An initial descriptor selection based on the knowledge of the reaction mechanism is followed by a mathematical screening where possible combinations of the selected descriptors are tried on the whole dataset. The relevance of each descriptor to the target activity is revealed in terms of the magnitude and sign of the respective coefficients. Models with higher *R*^2^ and lower SD values are selected for further use, while the rest are rejected. The selected combinations of descriptors are then employed on a training–test split of the dataset. The model is trained on the training set and is used to predict the activity of the compounds in the test set. Sometimes a validation set is also included. The regression model is generated on the training set with the experimental toxicity (pLC_50_ or pIGC_50_) or bioactivity as the dependent and the computed descriptors as the independent variables, followed by utilizing it to predict the activity of the test set compounds.

The above approach of splitting the data has the drawback that its SD may vary depending on which compounds are placed in the training and test sets. To remove any such bias, the model needs to be fitted several times with different combinations of training–test pairs each time, followed by evaluating the statistical metrics of the test set and averaging them out. This technique is known as cross-validation. Threefold cross-validation is employed by splitting the dataset into three equi-sized groups (sets A, B, and C), where two of them form the training set while the other forms the test set. Three such combinations are obtained. QSAR models are generated on all of them to obtain their respective *R*^2^ and SD values. Another specific case of cross-validation, known as the leave-one-out (LOO) cross-validation, is an exhaustive method that includes all possible combinations within the dataset. In this method, the dataset (of, say, *N* number of compounds) is split into a training set with *N* − 1 number of compounds, leaving only one compound for testing the trained model. This is repeated *N* number of times and the desired metric is averaged. However, being a very exhaustive process, it tends to be rather time-consuming and computationally expensive.

### 3.2. Neural Networks (NNs)

Apart from MLR, we have also utilized a supervised machine learning technique [85,86,87,88,89,90,91,92,93], the multilayer perceptron (MLP) neural network [53,54,94,95,96,97,98,99,100,101,102,103,104,105] for toxicological predictions by generating QSAR models with known values of hydrophobic (log*P*) and electronic (*ω*) parameters. An MLP comprises input, hidden, and output layers, where the units in the hidden layer are connected to those of the input layer by certain weights. The units in the output layer are connected in a similar fashion to those of the hidden layer. The hidden layer consists of a non-linear (most commonly, sigmoid) transfer function, y = 1/(1 + *e*^−*x*^), where *x* is the total weighted input for the unit. Such transfer functions allow the hidden layer to mimic bio-neurons, hence earning it the name “artificial neurons”. The output layer units also consist of transfer functions which vary depending on the desired application of the MLP, e.g., while sigmoid functions are preferred for classification problems, linear functions are better suited for regression problems to predict real-valued quantities (like pIGC_50_). 

In our studies, we have initialized all the weights with random values. The training data of descriptor-activity pairs are supplied as input, and the output error is calculated for each input as the squared difference of the calculated output from the real output. This error value is then back-propagated to update the weights between hidden-output and input-hidden layers. The process is repeated until convergence is reached, where the final set of weights is the trained set of parameters for the MLP which is then ready to predict the target activity of the unknown molecules in the test set. This step is done by supplying the descriptor values which get multiplied by the weights and sent to the hidden layer, where the sigmoid function acts on them to predict the target activity.

## 4. Case Studies

### 4.1. Pimephales Promelas

The toxicity of a set of 15 benzene derivatives (optimized structures are shown in Figure 1) towards *Pimephales promelas* (fathead minnow) is studied by developing SAR models with pLC_50_ as the dependent variable and the hydrophobicity and electrophilicity indices as the descriptors [56]. Initially, a training–test split of 10 and 5 is considered, followed by the threefold cross-validation where each subdivision of the dataset (i.e., A, B, and C) has five molecules each. The training was done taking either of the two sets, and then the test was performed with the third set.

**Figure 2:**
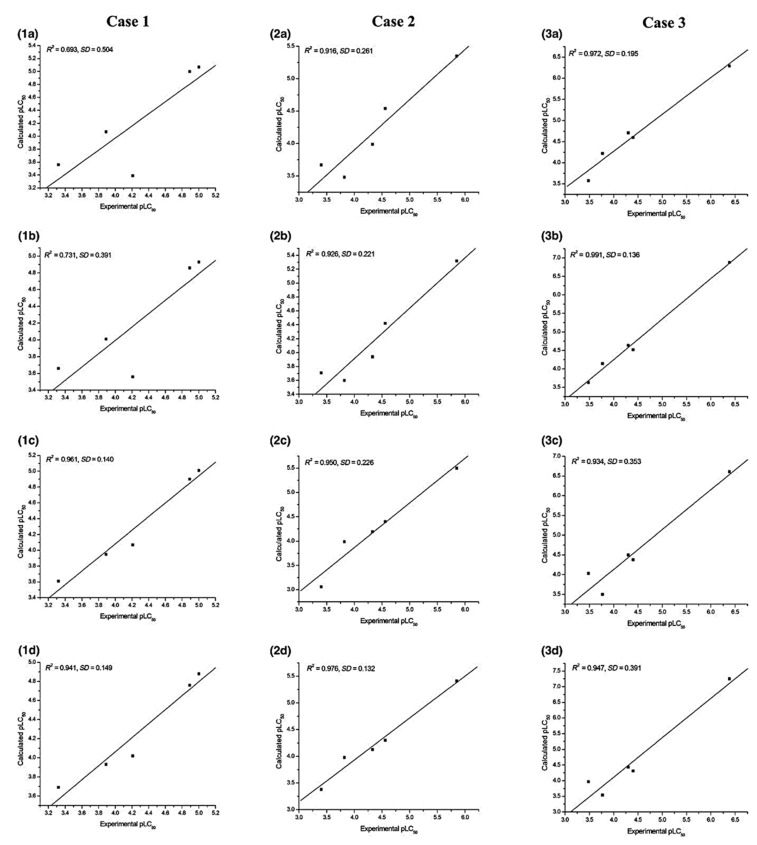
Experimental vs. calculated pLC_50_ plots with respect to (**a**) *ω*, (**b**) *ω*^2^, (**c**) log*P*, and (**d**) (log*P*)^2^, for the test set with regression models developed using the training set in all three cases of the three-fold CV (reprinted from ref. [56] with permission from John Wiley & Sons. © 2018 John Wiley & Sons A/S).

The prediction ability of the regression models generated is judged in terms of their *R*^2^, adjusted-*R*^2^, and *SD* values. Several combinations of the electrophilicity (*ω*, *ω*^2^, and *ω*^3^) and hydrophobicity (log*P* and (log*P*)^2^) are taken as the independent variables. The regression models obtained by applying the three-fold cross-validation technique reveal that *ω*^2^ (with an average *R*^2^ of 0.890) shows a higher correlation compared to that of *ω* (average *R*^2^ = 0.864) or *ω*^3^ (average *R*^2^ = 0.882). The results obtained considering log*P* and (log*P*)^2^ are comparable to those obtained by using the electrophilicity indices. Considering the fact that *ω* can be obtained much more easily through computation than obtaining the experimental values of log*P*, the former is more convenient, cheaper, and faster to use than the latter. The plot showing a good correlation between the experimental and predicted pLC_50_ values for different cases is provided in Figure 2. Analysis through the MLP neural network validates the results obtained from employing the simple MLR technique.

### 4.2. Tetrahymena Pyriformis

**Figure 3:**
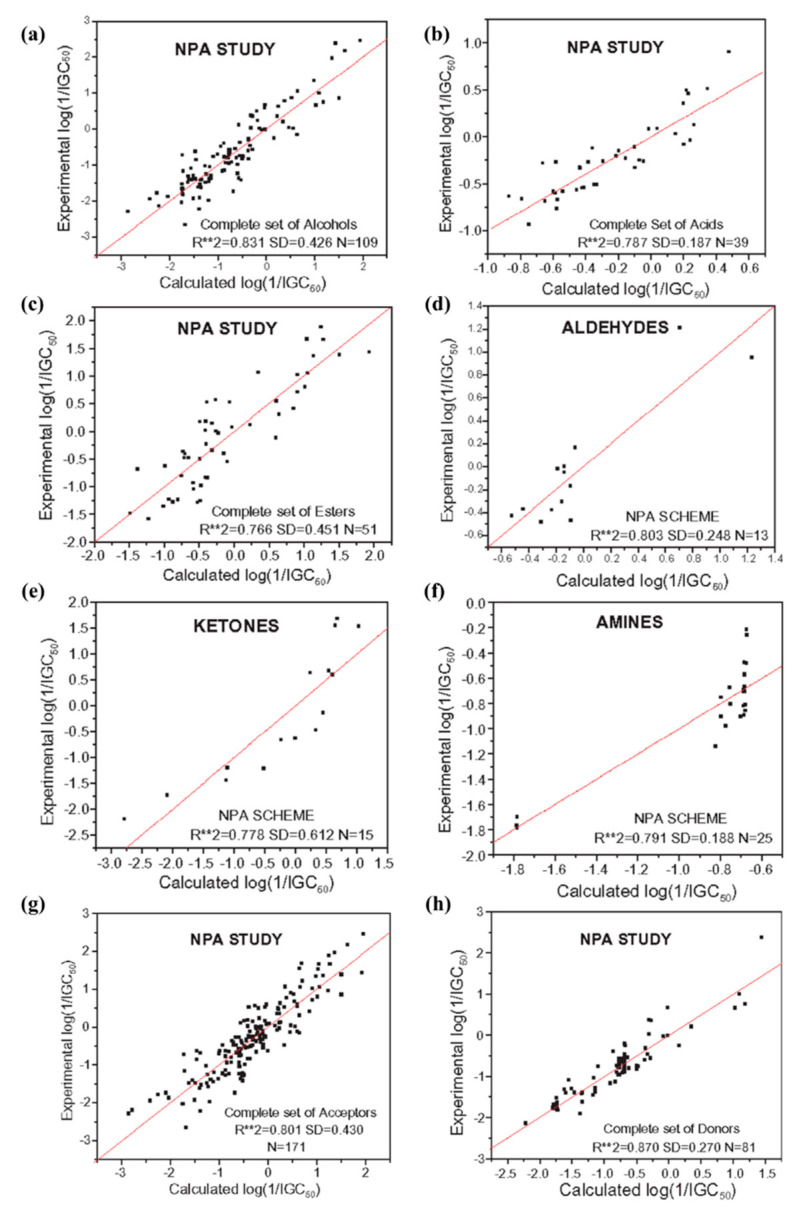
Correlation between the experimental and calculated log((IGC_50_)^−1^) of (**a**) alcohols, (**b**) esters, (**c**) acids, (**d**) aldehydes, (**e**) ketones, (**f**) amines, (**g**) all electron accepting compounds, and (**h**) all electron donating compounds (assorted from Ref. [78] Copyright © 2005 Elsevier Ltd.).

A total of 252 aliphatic compounds grouped into six different categories based on their primary functional group, viz., alcohols, esters, acids, aldehydes, ketones, and amines, are selected for studying their toxicity (log(IGC_50_^−1^)) against the ciliate *Tetrahymena pyriformis* [78]. CDFT-based electronic descriptors, electrophilicity, and local philicity are considered as the independent variables for the QSTR modeling for toxicity prediction. The study is performed for each group separately, and for the whole dataset as well. Each of the six aforementioned groups (and their respective subgroups) is designated as electron accepting or donating by comparing their electronegativity values with those of the nucleic acid bases/selected DNA base pairs. Depending on whether the compound has an electron accepting or donating tendency, the local philicity parameter, *ω*_m_^+^ or *ω*_m_^−^, respectively, is chosen as its descriptor of choice. To judge the model efficacy, the statistical parameter, viz., coefficient of determination (*R*^2^), is calculated. Figure 3 depicts the correlation between the experimental and calculated toxicity values of all the six aforementioned groups of compounds, along with those for the electron accepting and donating groups. *R*^2^ obtained in these cases are 0.831 (109 aliphatic alcohols), 0.787 (39 aliphatic acids), 0.766 (51 aliphatic esters), 0.803 (13 aliphatic aldehydes), 0.778 (15 ketones), and 0.791 (25 aliphatic amines). Similar calculations performed for all the electron acceptor compounds (171), irrespective of their functional group, delivers an *R*^2^ of 0.801. The same for all the electron donor compounds (81) is 0.870. From the correlations obtained, it is very clear that the global electrophilicity along with local philicity makes up a good pair of descriptors in explaining the toxicity of these aliphatic compounds against *T. pyriformis*, especially when previous studies [106,107,108,109] have reported SAR models with a higher number of predictors and/or with poor correlations. The toxicity of the entire dataset is analyzed in terms of the number of carbon atoms present in the respective compounds [110].

**Figure 4:**
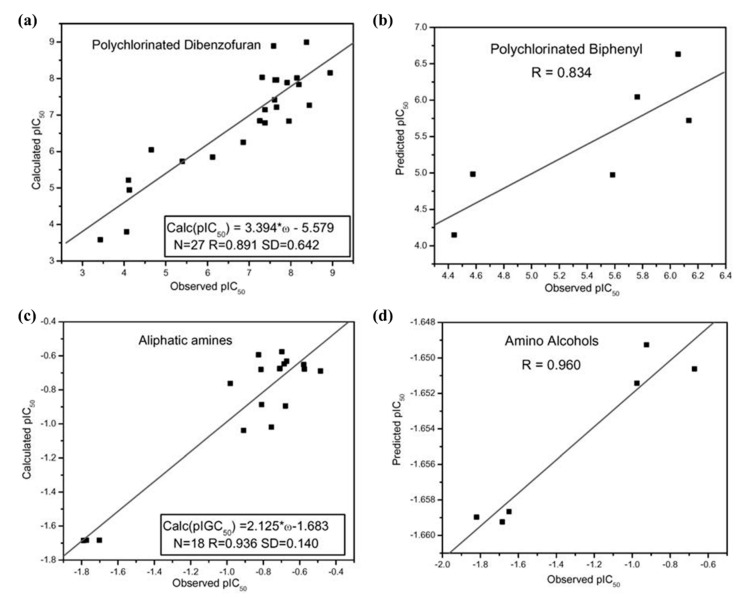
Plots of calculated vs. observed pIC_50_ for (**a**) training set of PCDFs, (**b**) test set of PCBs, (**c**) training set of aliphatic amines, and (**d**) test set of amino alcohols (adapted from Ref. [77] with permission from Springer Nature. © 2006, Springer Science Business Media, Inc.).

The global and local electrophilicity indices are also employed [77] on a dataset of polyaromatic hydrocarbons (PAH) to analyze the extent of their toxic effects towards displacing half of the radio-labeled tetrachlorodibenzo-*p*-dioxin (TCDD) from an arylhydrocarbon (Ah) receptor. For a dataset of electron acceptor [39,46] toxins, viz., polychlorinated dibenzofurans (PCDF), the model is trained using experimental pIC_50_ values as dependent and *ω* as the independent variables, respectively. The correlations obtained while using *ω* as the sole descriptor for the PCDFs, in the training–test split and the whole dataset, are *R* = 0.891 (for training; 0.834 for the test), and 0.891 (with an *R*^2^ of 0.786), respectively. An *F*-ratio of 96.743 is obtained, which is a good number for the given number of data points and compared to other parameters. Ponec’s [111] method is employed to calculate the probability of any random distribution producing the same *R*^2^, which turned out to be 10^−6^, ruling out the possibility of any chance correlation. The dataset of PCBs is then used as an external validation set to test the efficacy of the predictive ability of the QSAR model constructed for the training set of the PCDFs. A correlation of 0.834 is obtained for the same, suggesting the relevance of *ω* as the sole descriptor in such electrophile–nucleophile mechanism-driven processes (see Figure 4).

In the presence of biomolecules, while PCDFs and PCBs exhibit an electron-accepting nature [39,52,112,113], aliphatic amines behave as electron donors. The electron accepting/donating nature of these compounds is determined by analyzing the transfer of a fractional number of electrons (Δ*N*) from these compounds to the DNA base pairs/nucleic acid bases. A positive and negative Δ*N* represents the compounds as electron acceptors and donors, respectively. The toxicity of these amines towards *T. pyriformis* is also studied. They interact with the biomolecules through the donation of electrons [78]. Since there exists no reliable measure of global nucleophilicity, the maximum value of the local variant (*ω*_max_^−^) at the *N* atom of the amines is considered as the independent variable of the regression model. A correlation (*R*) of 0.936 was obtained with the NPA-derived *ω*_max_^−^ values for the QSAR equation:

(16) $$\mathrm{pIC}50=2.2137\;(0.2076)\;*\;\omega_{N\;\max}^{-}-1.6895(+0.0822)$$

*N* = 18, *SD* = 0.1493, *R* = 0.9363, *R*^2^_*adj*_ = 0.8689

A high *F*-ratio of 113.7066 is obtained, and a high *Q*^2^ value (leaving five points) of 0.8243 is obtained. The possibility of a chance correlation is ruled out by a probability value of 0.18 × 10^−5^. Further, models are also generated for the combined datasets of amines and amino alcohols, followed by a training–test split. The predictive ability of global and local electrophilicity indices is demonstrated for both electron acceptors and donors in both the gas and solution phases. 

Another study [114] on *T. pyriformis* includes the performance of an extensive MLP neural network and an MLR study using electrophilicity indices in conjunction with the hydrophobicity parameters of 169 aliphatic compounds. The correlation coefficient of the models based on the hydrophobicity and electrophilicity parameters lies within 0.790–0.983 and 0.703–0.779. Among all possible descriptor combinations, the best QSTR models turned out to be the ones with {*ω*, log*P*}, {*ω*^2^, log*P*} and {log*P*, (log*P*)^2^} pairs of descriptors. 

### 4.3. Trypanosoma Brucei 

A total of 32 numbers of pyridyl benzamide derivatives are considered for the study against the sleeping sickness, Human African trypanosomiasis (HAT) causing parasite *Trypanosoma brucei* [79]. All possible combinations of *ω*, *ω*^2^ and other descriptors utilized by Masand and coworkers [115] (provided in Table 1) are utilized to generate relevant regression models through MLR. The *R*^2^ and SD values obtained for the undivided dataset and the three combinations of the training–test split in the three-fold CV are provided in Table 2, which indicates that the 1st, 3rd, 4th, 6th, 7th, and 10th models show good correlation, while for the rest of the models the *R*^2^ does not cross the threshold value of 0.6 and is thus statistically irrelevant. An important observation is noted that on the removal of the RDF55s descriptor, there is a drastic reduction in the model efficiency, which clearly suggests its importance in this study.

**Figure 5:**
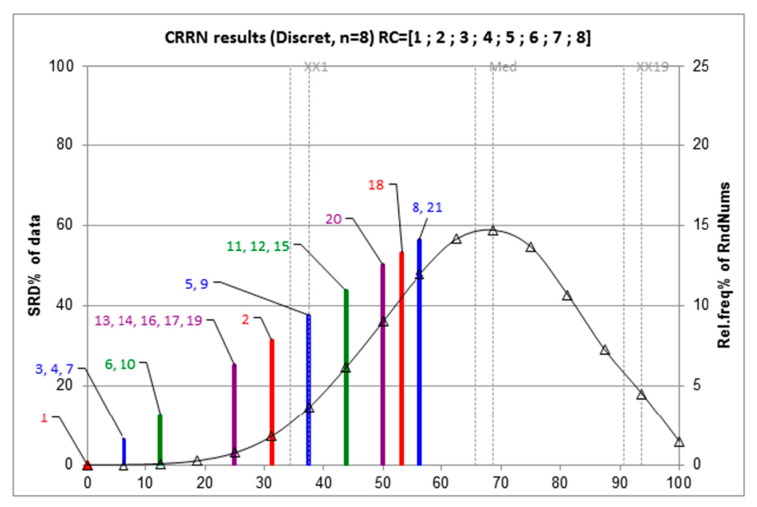
Descriptors choice evaluation using SRD with ties and a scaling between 0–100. The different colored lines represent ranking of different regression models, showcasing their relative position and extent of the similarity. The respective model numbers are provided as per Table 2. (reprinted from Ref. [79]. © 2019, IGI Global).

A technique for comparing the developed regression models, known as the sum of ranking differences (SRDs) [116,117], is carried out where the models are ranked in order of their efficacy. It requires the data to be arranged in a matrix structure with the rows representing the statistical metrics (*R*^2^ and SD), and the columns are the models to be ranked. An ideal or golden standard is chosen (here they are the highest *R*^2^ and least SD values) whose difference from the *R*^2^ and SD values of each of the models generates the SRD values. These SRD values are then compared to judge the models’ efficacy. In this ranking method, the lower the SRD value, the better is the model. Figure 5 represents the ranking of the models, showcasing their relative position and extent of the similarity.

Several other studies on QSAR modeling are reported on the effective use of ω as the electronic factor. The toxicity of arsenic ions is predicted using regression models based on atomic charge along with global and local electrophilicity indices. The model was trained on datasets of alkali and transition-metal ions [118]. The bio-activity of the derivatives of the sex hormones, testosterone and estrogen, are also successfully reported in terms of *ω* [40]. Several other quantum chemical descriptors, viz., IP, EA, *η*, softness (*S*), *χ*, along with *ω* are employed for studying the correlations in an alkane series, where it is noted that the IP performs best in describing various macroscopic properties [119]. 

## 5. Conclusions

The present article focuses on an analysis relating to the simplest yet effective regression techniques in the field of QSAR. Specifically, it delivers strong evidence for the effectiveness of both global and local electrophilicities in predicting toxicity and bioactivity. The ease of their computation compared to other common descriptors comes as an added bonus for their extensive usage. Employing them, either as solo descriptors in single-parameter models, or with other descriptors like the number of carbon atoms, charge transfer, hydrophobicity, etc., in multi-parameter regression models, has resulted in high coefficients of determination and low standard deviation. Needless to say, however, prior knowledge of the reaction mechanism is of the utmost importance in order to select appropriate descriptors for the target activity.

## Figures and Tables

**Table 1:** List of descriptors obtained from Ref. [115] used along with the electrophilicity index to develop QSTR models describing the HAT activity of pyridyl benzamide derivatives.

GATS8c	Geary autocorrelation of lag-8/weighted by atomic charges
RDF40p	Radial distribution function-040/weighted by relative polarizabilities
RDF55s	Radial distribution function-055/weighted by relative I-state
E1	1st component accessibility directional WHIM index/weighted by relative I-state
RDF40m	Radial distribution function-040/weighted by relative mass

**Table 2:** *R*^2^ and SD values obtained for the undivided dataset and the three combinations of training–test split in the three-fold cross-validation (reprinted from Ref. [79]. © 2019, IGI Global).

Model No.	Generalized Regression Equations	Undivided		Case 1		Case 2		Case 3	
		R^2^	SD	R^2^	SD	R^2^	SD	R^2^	SD
1	pIC_50_ = a + b*GATS8c + c*RDF40p + d*RDF55s	0.8284	0.1960	0.9182	0.1320	0.8216	0.1877	0.6971	0.2213
2	pIC_50_ = a + b*GATS8c + c*RDF40p + d*ω	0.3743	0.3742	0.4839	0.1872	0.3764	0.1546	0.2351	0.4378
3	pIC_50_ = a + b*GATS8c + c*ω + d*RDF55s	0.7599	0.2318	0.8325	0.1768	0.7829	0.2063	0.5114	0.2415
4	pIC_50_ = a + b*ω + c*RDF40p + d*RDF55s	0.7113	0.2542	0.7644	0.1983	0.7098	0.2283	0.4961	0.2529
5	pIC_50_ = a + b*GATS8c + c*RDF40p + d*ω^2^	0.3650	0.3770	0.4825	0.1827	0.3901	0.1447	0.2211	0.4098
6	pIC_50_ = a + b*GATS8c + c*ω^2^ + d*RDF55s	0.7592	0.2322	0.8323	0.1762	0.7853	0.2058	0.5817	0.2299
7	pIC_50_ = a + b*ω^2^ + c*RDF40p + d*RDF55s	0.7068	0.2562	0.7620	0.1977	0.7101	0.2301	0.4645	0.2575
8	pIC_50_ = a + b*GATS8c + c*ω + d*ω^2^	0.3285	0.3877	0.4334	0.1888	0.2506	0.1882	0.1725	0.4421
9	pIC_50_ = a + b*ω + c*RDF40p + d*ω^2^	0.3660	0.3767	0.4746	0.1781	0.2911	0.1800	0.1810	0.4594
10	pIC_50_ = a + b*ω + c*ω^2^ + d*RDF55s	0.6836	0.2661	0.7637	0.2014	0.6962	0.2212	0.5163	0.2364
11	pIC_50_ = a + b*E1s + c*RDF40m + d*GATS6m	0.3056	0.3942	0.3991	0.1983	0.3199	0.2404	0.1159	0.2055
12	pIC_50_ = a + b*E1s + c*RDF40m + d*ω	0.3647	0.3771	0.4949	0.1839	0.3196	0.1784	0.2793	0.3939
13	pIC_50_ = a + b*E1s + c*ω + d*GATS6m	0.4847	0.3396	0.5936	0.2275	0.5415	0.2138	0.3369	0.3171
14	pIC_50_ = a + b*ω + c*RDF40m + d*GATS6m	0.4758	0.3425	0.5824	0.2177	0.5019	0.2127	0.3263	0.3134
15	pIC_50_ = a + b*E1s + c*RDF40m + d*ω^2^	0.3571	0.3793	0.4997	0.1769	0.3241	0.1706	0.2135	0.4273
16	pIC_50_ = a + b*E1s + c*ω^2^ + d*GATS6m	0.4763	0.3424	0.5839	0.2266	0.5473	0.2083	0.2648	0.3481
17	pIC_50_ = a + b*ω^2^ + c*RDF40m + d*GATS6m	0.4666	0.3455	0.5708	0.2181	0.5105	0.2030	0.2518	0.3441
18	pIC_50_ = a + b*E1s + c*ω + d*ω^2^	0.3421	0.3421	0.4673	0.1865	0.2898	0.1777	0.1339	0.4681
19	pIC_50_ = a + b*ω + c*ω^2^ + d*GATS6m	0.4784	0.3417	0.5861	0.2301	0.4044	0.2680	0.1784	0.3648
20	pIC_50_ = a + b*ω + c*RDF40m + d*ω^2^	0.3583	0.3790	0.4822	0.1844	0.2185	0.2091	0.1454	0.4405
21	pIC_50_ = a + b*ω + c*ω^2^	0.3272	0.3813	0.4540	0.1813	0.2922	0.1765	0.1288	0.4612

## Data Availability

Data is contained within the article and supplementary material.

## Supplementary Materials

The following supporting information can be downloaded at: https://www.mdpi.com/article/10.3390/ph15111383/s1, Table S1: Polychlorinated dibenzofurans with identity number (ID) representing the substitution pattern. (reprinted from ref. [77] with permission from Springer Nature. Copyright © 2006, Springer Science Business Media, Inc.); Table S2: Polychlorinated biphenyls with identity number (ID) rep- resenting the substitution pattern. (reprinted from ref. [77] with permission from Springer Nature. Copyright © 2006, Springer Science Business Media, Inc.). Table S3: Dataset of 252 aliphatic compounds considered against *Tetrahymena pyriformis* [78]. Table S4: The dataset of 32 pyridyl benzamides considered against *Trypanosoma brucei* (reprinted from Ref. [79]. © 2019, IGI Global).
