# Supplementary material for: Quantitative Structure–Toxicity Relationship in Bioactive Molecules from a Conceptual DFT Perspective
Source: Pharmaceuticals (Basel). 2022 Nov 10;15(11):1383. doi: 10.3390/ph15111383 (PMC9695291; doi:10.3390/ph15111383)
Supplement: Supplementary file 1 [file pharmaceuticals-15-01383-s001.zip › pharmaceuticals-1969517-supplementary.pdf]

Quantitative structure–toxicity relationship in bioactive molecules from a conceptual DFT perspective

Ranita Pal,[1] Shanti Gopal Patra,[2] and Pratim Kumar Chattaraj[2,*]

[1]*Advanced Technology Development Centre, Indian Institute of Technology Kharagpur, 721302, India.*

[2]*Department of Chemistry, Indian Institute of Technology Kharagpur, West Bengal - 721302, India*

* Corresponding author: pkc@chem.iitkgp.ac.in (PKC) (ORCID; 0000-0002-5650-7666)

SUPPLIMENTARY INFORMATION

Table S1: Polychlorinated dibenzofurans with identity number (ID) representing the substitution pattern. (reprinted from ref. [77] with permission from Springer Nature. Copyright © 2006, Springer Science Business Media, Inc.)

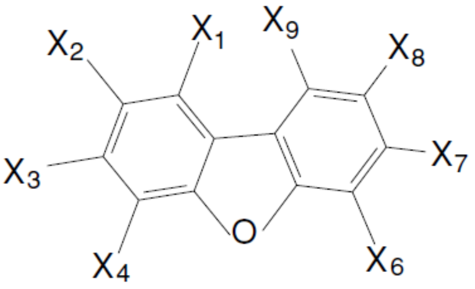

ID	X_1	X_2	X_3	X_4	X_6	X_7	X_8	X_9
1	H	Cl	H	H	H	H	H	H
2	H	H	H	Cl	H	H	H	H
3	H	Cl	H	H	Cl	H	H	H
4	H	Cl	H	H	H	H	Cl	H
5	Cl	H	Cl	H	Cl	H	H	H
6	Cl	H	Cl	H	H	H	Cl	H
7	H	Cl	Cl	Cl	H	H	H	H
8	H	Cl	Cl	H	H	H	Cl	H
9	H	Cl	H	H	Cl	Cl	H	H
10	H	Cl	Cl	Cl	Cl	H	H	H
11	H	Cl	Cl	Cl	H	H	Cl	H
12	H	Cl	Cl	H	H	Cl	Cl	H
13	Cl	Cl	H	Cl	Cl	Cl	H	H
14	Cl	Cl	Cl	Cl	H	H	Cl	H
15	Cl	Cl	Cl	H	H	Cl	Cl	H
16	H	Cl	Cl	Cl	H	Cl	Cl	H

17	Cl	Cl	Cl	Cl	H	Cl	Cl	H
18	H	Cl	Cl	Cl	Cl	Cl	Cl	H
19	H	Cl	Cl	H	Cl	H	Cl	H
20	Cl	Cl	Cl	H	Cl	H	H	H
21	Cl	Cl	Cl	H	H	Cl	H	H
22	Cl	H	Cl	Cl	H	Cl	Cl	H
23	H	Cl	Cl	Cl	H	Cl	H	Cl
24	Cl	Cl	Cl	H	H	Cl	H	Cl
25	Cl	Cl	Cl	H	H	Cl	H	H
26	Cl	H	Cl	Cl	H	Cl	Cl	H
27	H	Cl	Cl	Cl	H	Cl	H	Cl

Table S2: Polychlorinated biphenyls with identity number (ID) rep-resenting the substitution pattern. (reprinted from ref. [77] with permission from Springer Nature. Copyright © 2006, Springer Science Business Media, Inc.)

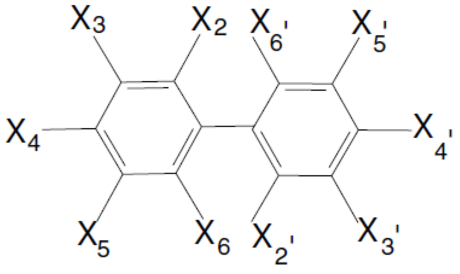

ID	X_2	X_3	X_4	X_5	X_6	$X_{2'}$	$X_{3'}$	$X_{4'}$	$X_{5'}$	$X_{6'}$
28	Cl	H	H	H	H	H	Cl	Cl	Cl	H
29	Cl	Cl	Cl	H	H	H	Cl	Cl	H	H
30	Cl	H	Cl	Cl	H	H	Cl	Cl	H	H
31	Cl	Cl	Cl	Cl	H	H	Cl	Cl	H	H
32	Cl	H	Cl	H	H	Cl	H	Cl	H	H
33	Cl	H	Cl	H	Cl	H	Cl	Cl	Cl	H

Table S3: Dataset of 252 aliphatic compounds considered against *Tetrahymena pyriformis*. [78]

	Alcohols: amino alcohols		***Saturated alcohols***	23	1-Hexen-3-ol
1	2-(Methylamino)ethanol	1	Methyl alcohol	24	*cis*-2-Hexen-1-ol
2	4-Amino-1-butanol	2	Ethyl alcohol	25	*trans*-2-Octen-1-ol
3	2-(Ethylamino)ethanol	3	1-Propanol		***Acids: carboxylic acids***
4	2-Propylaminoethanol	4	2-Propanol	1	Propanoic acid
5	DL-2-Amino-1-pentanol	5	1-Butanol	2	Butyric acid
6	3-Amino-2,2-dimethyl-1-propanol	6	(±)-2-Butanol	3	Valeric acid
7	6-Amino-1-hexanol	7	2-Methyl-1-propanol	4	Hexanoic acid
8	DL-2-Amino-1-hexanol	8	2-Pentanol	5	Heptanoic acid
9	DL-2-Amino-3-methyl-1-butanol	9	3-Pentanol	6	Octanoic acid
10	2-Amino-3,3-dimethyl-butanol	10	3-Methyl-2-butanol	7	Nonanoic acid
11	2-Amino-3-methyl-1-pentanol	11	tert-Amylalcohol	8	Decanoic acid
12	2-Amino-4-methyl-pentanol	12	2-Methyl-1-butanol	9	Undecanoic acid
13	2-(*tert*-Butylamino)ethanol	13	3-Methyl-1-butanol	10	*iso*-Butyric acid
14	Diethanolamine	14	2,2-Dimethyl-1-propanol	11	Isovalerianic acid
15	1,3-Diamino-2-hydroxy-propane	15	2-Methyl-2-propanol	12	Trimethylacetic acid
16	*N*-Methyldiethanol amine	16	1-Hexanol	13	3-Methylvaleric acid
17	3-(Methylamino)-1,2-propanediol	17	3,3-Dimethyl-1-butanol	14	4-Methylvaleric acid
18	Triethanolamine	18	4-Methyl-1-pentanol	15	2-Ethylbutyric acid
	α-Acetylenic alcohols	19	1-Heptanol	16	2-Propylpentanoic acid
1	3-Butyn-2-ol	20	2,4-Dimethyl-3-pentanol	17	2-Ethylhexanoic acid
2	1-Pentyn-3-ol	21	1-Octanol	18	Succinic acid
3	2-Pentyn-1-ol	22	2-Octanol	19	Glutaric acid
4	2-Penten-4-yn-1-ol	23	3-Octanol	20	Adipic acid
5	1-Hexyn-3-ol	24	1-Nonanol	21	Pimelic acid
6	1-Heptyn-3-ol	25	2-Nonanol	22	3,3-Dimethylglutaric acid
7	4-Heptyn-3-ol	26	3-Ethyl-2,2-dimethyl-3-pentanol	23	Suberic acid
8	2-Octyn-1-ol	27	1-Decanol	24	Sebacic acid
9	2-Nonyn-1-ol	28	(±)-4-Decanol	25	1,10-Decanedicarboxylic acid
10	2-Decyn-1-ol	29	3,7-Dimethyl-3-octanol	26	Crotonic acid
11	2-Tridecyn-1-ol	30	1-Undecanol	27	*trans*-2-Pentenoic acid
12	4-Methyl-1-pentyn-3-ol	31	1-Dodecanol	28	*trans*-2-Hexenoic acid
13	4-Methyl-1-heptyn-3-ol	32	1-Tridecanol		***Halogenated acids***
	Diols		***Unsaturated alcohols***	1	4-Bromobutyric acid
1	(±)-1,2-Butanediol	1	2-Methyl-3-buten-2-ol	2	5-Bromovaleric acid
2	(±)-1,3-Butanediol	2	4-Pentyn-1-ol	3	4-Chlorobutyric acid
3	1,4-Butanediol	3	2-Methyl-3-butyn-2-ol	4	3-Chloropropionic acid
4	1,2-Pentanediol	4	*trans*-3-Hexen-1-ol	5	5-Chlorovaleric acid
5	1,5-Pentanediol	5	*cis*-3-Hexen-1-ol	6	2-Bromobutyric acid
6	2-Methyl-2,4-pentanediol	6	5-Hexyn-1-ol	7	2-Bromoisobutyric acid
7	(±)-1,2-Hexanediol	7	3-Methyl-1-pentyn-3-ol	8	2-Bromoisovaleric acid
8	1,6-Hexanediol	8	4-Hexen-1-ol	9	2-Bromovaleric acid
9	1,2-Decanediol	9	5-Hexen-1-ol	10	2-Bromooctanoic acid
10	1,10-Decanediol	10	4-Pentyn-2-ol	11	2-Bromohexanoic acid
	Halogenated alcohols	11	5-Hexyn-3-ol		***Esters: monoesters***
1	2-Bromoethanol	12	3-Heptyn-1-ol	1	Ethyl acetate
2	2-Chloroethanol	13	4-Heptyn-2-ol	2	Propyl acetate
3	1-Chloro-2-propanol	14	3-Octyn-1-ol	3	Isopropyl acetate
4	3-Chloro-1-propanol	15	3-Nonyn-1-ol	4	Butyl acetate
5	4-Chloro-1-butanol	16	2-Propen-1-ol	5	Amyl acetate
6	3-Chloro-2,2-dimethyl-1-propanol	17	2-Buten-1-ol	6	Hexyl acetate
7	6-Chloro-1-hexanol	18	(±)-3-Buten-2-ol	7	Octyl acetate
8	8-Chloro-1-octanol	19	*cis*-2-Buten-1,4-diol	8	Decyl acetate
9	6-Bromo-1-hexanol	20	*cis*-2-Penten-1-ol	9	Ethyl propionate
10	8-Bromo-1-octanol	21	3-Penten-2-ol	10	Butyl propionate
11	2,3-Dibromopropanol	22	*trans*-2-Hexen-1-ol	11	Isobutyl propionate

(Table S3 continued…)

	***Esters: monoesters** (continued)*	12	Diethyl pimelate	10	2-Nonanone
12	Propyl propionate	13	Dibutyl suberate	11	2-Decanone
13	*tert*-Butyl propionate	14	Diethyl butylmalonate	12	3-Decanone
14	Ethyl butyrate	15	Diethyl ethylmalonate	13	2-Undecanone
15	Ethyl isobutyrate	16	Diethyl 3-oxopimelate	14	2-Dodecanone
16	Ethyl valerate	17	Diethyl 4-oxopimelate	15	7-Tridecanone
17	Propyl butyrate	18	Diethyl methylmalonate		***Amines***
18	Butyl butyrate	19	Diethyl propylmalonate	1	Propylamine
19	Propyl valerate	20	Dibutyl succinate	2	Butylamine
20	Amyl propionate		***Aldehydes***	3	*N*-Methylpropylamine
21	Ethyl hexanoate	1	Propionaldehyde	4	Amylamine
22	Methyl butyrate	2	Butyraldehyde	5	*N*-Methylbutylamine
23	Methyl valerate	3	Isobutyraldehyde	6	Hexylamine
24	Methyl hexanoate	4	Valeraldehyde	7	Isopropylamine
25	Methyl heptanoate	5	2-Methyl-butyraldehyde	8	Isobutylamine
26	Methyl octanoate	6	Hexylaldehyde	9	*N*,*N*-Dimethylethylamine
27	Methyl nonanoate	7	2-Methylvaleraldehyde	10	(±)-*sec*-Butylamine
28	Methyl decanoate	8	2-Ethylbutyraldehyde	11	Isoamylamine
29	Methyl undecanoate	9	3,3-Dimethylbutyraldehyde	12	1-Methylbutylamine
30	Methyl formate	10	Heptaldehyde	13	1-Ethylpropylamine
31	*tert*-Butyl formate	11	2-Ethylhexanal	14	2-Methylbutylamine
	Diesters	12	*trans*-4-Decen-1-al	15	*N*,*N*-Diethylmethylamine
1	Diethyl malonate	13	*cis*-7-Decen-1-al	16	*tert*-Butylamine
2	Diethyl sebacate		***Ketones***	17	*tert*-Amylamine
3	Diethyl suberate	1	Acetone	18	(±)-1,2-Dimethylpropylamine
4	Diethyl succinate	2	2-Butanone	19	Propargylamine
5	Dimethyl malonate	3	2-Pentanone	20	N-Methylpropargylamine
6	Dibutyl adipate	4	3-Pentanone	21	1-Dimethylamino-2-propyne
7	Dimethyl succinate	5	4-Methyl-2-pentanone	22	1,1-Dimethylpropargylamine
8	Diethyl adipate	6	2-Heptanone	23	2-Methoxyethylamine
9	Dimethyl brassylate	7	5-Methyl-2-hexanone	24	3-Methoxypropylamine
10	Dimethyl sebacate	8	4-Heptanone	25	3-Ethoxypropylamine
11	Dimethyl suberate	9	2-Octanone		

Table S4: The dataset of 32 pyridyl benzamides considered against *Trypanosoma brucei* (reprinted from Ref. [79]. © 2019, IGI Global)

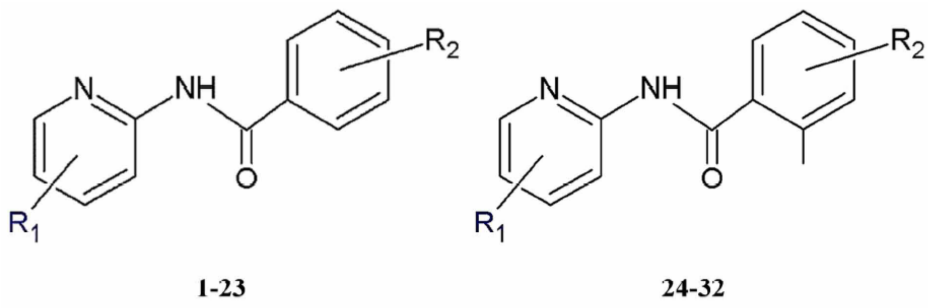

Sl. No.	R_1	R_2	Expt. pIC_{50}	ω	ω^2
1	H	2-Me	5.5190	0.37	0.14
2	4-CN	2-Me	5.6380	0.60	0.36
3	4-Me	2-Me	5.6780	0.36	0.13
4	4-Cl	2-Me	5.6780	0.44	0.19
5	4-Br	2-Me	5.7450	0.44	0.19
6	4-F	2-Me	6.2920	0.43	0.18
7	4-C≡CPh	2-Me	5.7700	0.47	0.22
8	4-C≡CCH_2-iPr	2-Me	5.9590	0.40	0.16
9	4-Ph	2-Me	5.6990	0.40	0.16
10	5-OMe	2-Me	5.4200	0.35	0.12
11	6-CH=$CH(CH_2)_2CH_3$	2-Me	5.2220	0.31	0.10
12	6-NH_2	2-Me	5.3370	0.30	0.09
13	H	H	5.0420	0.40	0.16
14	H	2-Et	5.5620	0.36	0.13
15	H	2-Me, 3-F	6.0510	0.41	0.17
16	H	3-F	5.1350	0.46	0.21
17	H	2-Me, 3-Cl	6.2010	0.40	0.16
18	H	2-Me, 3-Br	5.2440	0.41	0.16
19	H	2,3-diMe	5.4560	0.34	0.11
20	H	2-Me, 4-F	5.7210	0.38	0.15
21	H	2-Me, 4-Cl	5.9590	0.43	0.19
22	H	2-Me, 4-Br	5.9590	0.44	0.19
23	H	2,4-diMe	6.0090	0.36	0.13
24	H	3-F, 4-F	6.3870	0.47	0.22
25	4-Me	3-F	6.0810	0.48	0.23
26	4-Me	3-F, 4-F	6.0460	0.46	0.21
27	4-Cl	3-F	6.3280	0.53	0.28
28	4-Cl	3-F, 4-F	6.2760	0.54	0.30
29	4-F	3-F	7.0000	0.52	0.27
30	4-F	3-F, 4-F	6.7210	0.53	0.29
31	5-OMe	3-F	5.6380	0.43	0.19
32	5-OMe	4-F	5.5850	0.39	0.15
